# Association between *MTHFD1* G1958A Polymorphism and Neural Tube Defects Susceptibility: A Meta-Analysis

**DOI:** 10.1371/journal.pone.0101169

**Published:** 2014-06-30

**Authors:** Jianxin Jiang, Yanfei Zhang, Liang Wei, Zhiyang Sun, Zhongmin Liu

**Affiliations:** 1 Research Center for Translational Medicine, East Hospital, Tongji University School of Medicine, Shanghai, China; 2 Department of Neurosurgery, East Hospital, Tongji University School of Medicine, Shanghai, China; National Cancer Center, Japan

## Abstract

**Objectives:**

The methylenetetrahydrofolate dehydrogenase (*MTHFD1*) gene, as one of the key genes involved in the folate pathway, has been reported to play a critical role in the pathogenesis of neural tube defects (NTDs). However, the results of published studies are contradictory and inconclusive. Thus, this meta-analysis aimed to evaluate the effect of the common polymorphism in the *MTHFD1* gene, the G1958A (R653Q, dbSNP ID: rs2236225) variant, on the risk of NTDs in all eligible studies.

**Methods:**

Relevant literature published before January 3, 2014 was retrieved from the MEDLINE, EMBASE, Cochrane Library, and CBM databases. Pooled crude odds ratios (ORs) and their corresponding 95% confidence intervals (CIs) were calculated to evaluate the association between the *MTHFD1* G1958A polymorphism and NTDs risk.

**Results:**

We performed a meta-analysis of nine studies with a total of 4,302 NTDs patients and 4,238 healthy controls. Our results demonstrated a significant correlation between the *MTHFD1* G1958A polymorphism and NTDs in an overall meta-analysis. For family-based studies, the study subjects were classified as NTD cases, mothers with NTDs offspring, and fathers with NTDs offspring. We found no association between any of the fathers’ genotypes and NTDs, whereas there was a clear excess of the 1958A allele in the mothers of children with NTDs compared with controls individuals.

**Conclusions:**

In summary, our meta-analysis strongly suggests that the *MTHFD1* G1958A polymorphism might be associated with maternal risk for NTDs in Caucasian populations. However, the evidence of this association should be interpreted with caution due to the selective nature of publication of genetic association studies.

## Introduction

Neural tube defects (NTDs) are among the most common congenital malformations at birth and present as a wide range of phenotypes, primarily including anencephaly, spina bifida and encephalocele [1]. Approximately 1–2 in 1000 pregnancies worldwide are affected by NTD, but this rate varies between different populations and socio-economic groups [2]. The aetiology of NTDs is assumed to be multifactorial, with a large number of unclear genetic components, environmental conditions, and their interactions playing critical roles [3]. Epidemiological studies have revealed that periconceptional vitamin supplementation with folic acid substantially lowers the percentage of women with NTD-affected pregnancies [4], [5], which has led to intense research on the genetic variants of enzymes involved in the folate metabolic pathway. It is hypothesized that polymorphisms within folate-dependent enzymes might have an impact on NTDs risk, although the exact mechanism of these genetic factors has not been completely elucidated.

Numerous investigations of genes that are specifically involved in folate metabolism have identified at least one polymorphism, C677T (A222V; dbSNP ID: rs1801133) in the 5, 10-methylenetetrahydrofolate reductase (*MTHFR*) gene, that may be associated with an approximately doubled risk of NTDs [6]–[10]. Recently, a meta-analysis involving 25 case-control studies on the association between NTDs risk and the *MTHFR* 677TT genotype arrived at a pooled OR of 2.02 (95% CI 1.51–2.71) [11]. Nevertheless, the presence of this principal *MTHFR* variant does not appear to account for a large proportion of the etiologic factors for NTDs [12], indicating that besides *MTHFR*, additional folate-related genes are likely to also be involved.

Another folate-dependent enzyme, 5, 10-methylenetetrahydrofolate dehydrogenase (*MTHFD1*), given its central role in folate metabolism, is an attractive candidate gene in NTDs aetiology. *MTHFD1* is a nicotinamide adenine dinucleotide phosphate (NADP)-dependent trifunctional cytoplasmic enzyme (often referred to as “C_1_-THF synthase”), acting as 10-formyl, 5, 10-methenyl, and 5, 10-methylene derivatives [13], which plays an important role in folate metabolism. In addition to its enzymatic activity, biochemical evidence also demonstrates that *MTHFD1* plays a critical role as a structural component in methionine synthesis and *de novo* purine and pyrimidine synthesis [14]. Several potential single nucleotide polymorphisms (SNPs) in the *MTHFD1* gene were identified from public databases. Among them, a SNP at nucleotide 1958 of the *MTHFD1* gene, which causes a G to A transition (G1958A; dbSNP ID: rs2236225) that results in replacement of the arginine residue at position 653 by glutamine (R653Q), is one of the most attractive and frequently studied polymorphisms.

Initially, Hol et al. first investigated the association between *MTHFD1* 1958G>A and susceptibility to familial and sporadic NTDs in a Dutch population but failed to provide evidence for a major role of this alteration in NTDs etiology [15]. However, in subsequent studies conducted in an Irish population, both Brody et al. and Parle-McDermott et al. concluded that the *MTHFD1* G1958A polymorphism is associated with an increase in the genetically determined risk that a woman will bear a child with NTDs [16], [17]. De Marco et al. also observed a significant effect of the G1958A polymorphism on susceptibility to NTDs in an Italy population [18], whereas van der Linden et al. found no major risk associated with spina bifida in a Dutch population [19]. Considering the limited power of individual studies with small sample sizes, the association of the *MTHFD1* G1958A polymorphism with NTDs remains controversial and needs to be fully validated. To solve the problem of inadequate statistical power and controversial results, we performed a meta-analysis using published data from observational studies to provide empirical evidence on the association. To the best of our knowledge, this is the first meta-analysis to assess the association of the *MTHFD1* G1958A polymorphism with NTDs risk.

## Materials and Methods

To ensure the rigour of this meta-analysis, we designed and conducted it according to the guidelines of the Preferred Reporting Items for Systematic Reviews and Meta-analyses (PRISMA) (Supplement S1).

### Publication search

A comprehensive literature search for studies reporting on the association of the *MTHFD1* G1958A polymorphism with susceptibility to NTDs was conducted in the MEDLINE, EMBASE, Cochrane Library, CBM (Chinese Biomedical Literature) and CNKI (China National Knowledge Infrastructure) databases. The following combinations of MeSH terms and keywords were used: (‘neural tube defects’ or ‘anencephaly’ or ‘encephalocele’ or ‘meningomyelocele’ or ‘spinal dysraphism’ or ‘spina bifida occulta’ or ‘spina bifida cystica’) and (‘polymorphism’ or ‘single nucleotide polymorphisms’ or ‘polymorphism, genetic’) and (‘methylenetetrahydrofolate dehydrogenase’ or ‘MTHFD1’ or ‘G1958A’ or ‘rs2236225’ or ‘R653Q’). A manual search was also carried out to find additional potential studies in the references lists of reviews. The latest search was performed on January 3, 2014 without any limitation on language.

### Validity assessment

The following inclusion criteria were used to select potential studies for this meta-analysis: (1) evaluated the relationship between the *MTHFD1* G1958A polymorphism and susceptibility to NTDs; (2) all patients in the candidate studies met the diagnostic criteria for NTDs and controls were without NTDs; and (3) sufficient available genotype data for estimating ORs with their corresponding 95%CIs. The major reasons for the exclusion of studies are as follows: (1) not related to the G1958A polymorphism and NTDs; (2) repeated or duplicate studies; (3) studies only examining case populations; and (4) no usable data reported. When there were multiple studies with the same or overlapping data, only the most recent one with the most subjects was selected for this meta-analysis.

### Data extraction and quality assessment

Following the PRISMA guide, two investigators independently reviewed and checked all full-text reports and extracted relevant information, including trial features (e.g., surname of first author, year of publication and country of origin), participants’ general features (e.g., ethnicity, definition and number of case/control subjects), genotyping methods, data needed for meta-analysis (e.g., the frequencies of the alleles and the genotypic distributions for both the cases and controls), evidence of Hardy-Weinberg equilibrium (HWE) in controls, etc. Discrepancies were solved through discussion until consensus was reached. When crucial data was not reported in the original publication, required information was obtained by contacting the authors when possible.

The methodological quality of all included studies was assessed by the Newcastle-Ottawa Scale (NOS) criteria (Supplement S2) [20]. The NOS criteria is used to analyze the study quality with regard to selection, comparability, and exposure. Scores range from 0 stars (worst) to 9 stars (best), with a score of 5 or higher indicating a moderate-high methodological quality. Any disagreements over quality scores were resolved by discussion and subsequent consensus.

### Statistical analysis

All statistical analyses were performed using the STATA software (version 12.0, Stata Corp., College Station, TX, USA). And two-tailed *P*<0.05 was considered statistically significant. Genotype frequencies of the *MTHFD1* G1958A polymorphism in the control groups were tested for conformation to HWE using the chi-square test. The strength of the association between the *MTHFD1* G1958A polymorphism and susceptibility to NTDs was assessed by the pooled ORs, whereas a sense of the precision of the estimate was provided by their corresponding 95% CIs. We examined *MTHFD1* G1958A genotypes using five genetic models, including allele (A allele vs. G allele), dominant (AA+AG vs. GG), recessive (AA vs. AG+GG), homozygous (AA vs. GG), and heterozygous (AA vs. AG) comparisons. The significance of the pooled ORs was determined by the *Z*-test with a *P*-value less than 0.05 indicating statistical significance.

Taking into consideration possible heterogeneity between-studies, Cochran’s Q statistic and the *I^2^* metric were conducted [21], [22]. Values of *P* less than 0.10 and *I^2^* exceeding 50% were considered to indicate the presence of significant heterogeneity. Either a random-effects (DerSimonian-Laird method) or fixed-effects model (Mantel-Haenszel method) was applied to calculate pooled effect estimates in the presence or absence of heterogeneity, respectively. In addition, to assess possible publication bias, the Begg’s funnel plot and Egger’s liner regression test were conducted [23], [24]. Sensitivity analyses were performed by omitting individual studies in turn to determine if any studies significantly affected the original results [25]. A subgroup analysis of the family-based studies on different populations (NTDs children, mothers with NTDs offspring, and fathers with NTDs offspring) was also performed.

## Results

### Study characteristics

**Figure 1:**
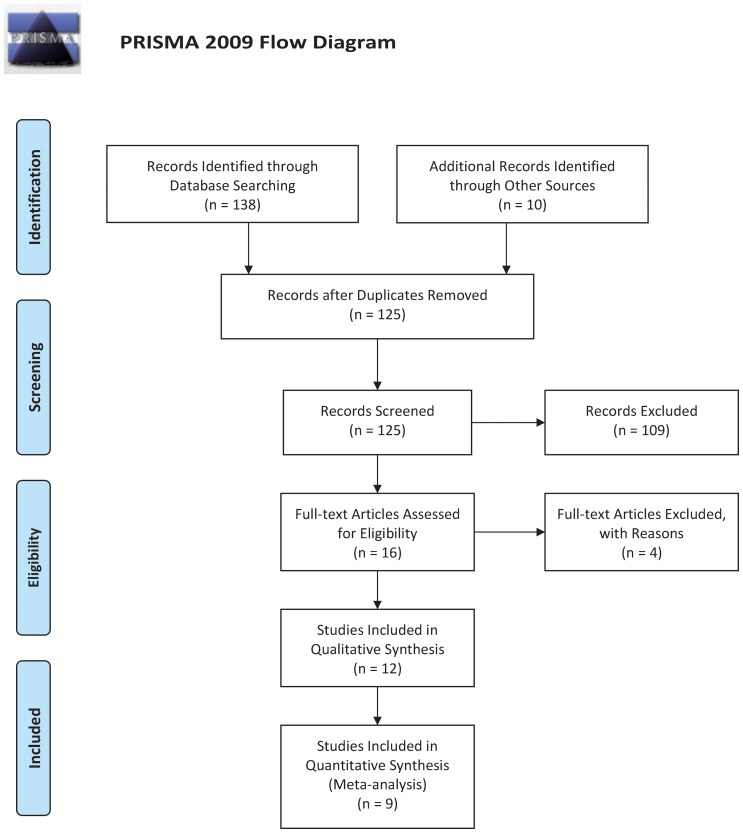
Flow diagram of studies with specific reasons for inclusion/exclusion in the present meta-analysis.

Figure 1 presents a flow chart for the process of study retrieval and exclusion. Based on the pre-specified search strategy and inclusion criteria, a total of 148 potentially references were preliminarily identified. After removing duplicate records (n = 23) and articles without full texts (n = 10), 115 titles and abstract were reviewed; of these, 99 articles were excluded for the following reasons: 55 were not case-control studies, 16 were not relevant to NTDs, and 28 were not relevant to the *MTHFD1* G1958A polymorphism. Thus, 16 articles were retained for a full text evaluation. Among them, 4 articles were excluded for including irrelevant genotypic and allelic frequency data (Supplement S3). Finally, 12 studies included in qualitative synthesis [15]–[19], [26]–[32], and nine studies were included in quantitative analysis (meta-analysis) [15]–[19], [29]–[32], providing data on a total of 8,360 Caucasians (4,302 NTDs patients and 4,328 healthy controls). Of the included studies, five studies investigated the association of the *MTHFD1* G1958A polymorphism in NTDs cases [16]–[19], [29], five in mothers with NTDs offspring [16]–[19], [29] and four in fathers with NTDs offspring [16]–[18], [29]. NTDs cases with a wide range of severity (e.g. spina bifida, anencephaly, and encephalocele) were included in four studies, whereas only spina bifida cases were selected by van der Linden et al. [19], Carroll et al. [29], Shaw et al. [30], and Marini et al [31]. All studies in this meta-analysis were in HWE, except for two studies [17], [29]. The polymerase chain reaction (PCR) followed by restriction fragment length polymorphism (RFLP) analysis was the genotyping method used in all studies, besides Hol et al.’s study, which used the selected PCR-single strand conformation polymorphism (SSCP) method [15]. The main characteristics of all included studies are shown in Table 1.

**Table 1:** Main characteristics of the studies included in the meta-analysis.

Included studies	Country	Ethnicity	Sample size		Type of case/control subjects	Detection method	*P* HWE for controls	NOS star
			Case	Control				
Hol FA, 1998	Netherlands	Caucasian	103	335	Familial and sporadic NTD cases, healthy controls	PCR-SSCP	0.469	7/9
Brody LC, 2002	Ireland	Caucasian	1056	997	NTD children, NTD fathers, NTD mothers, female controls	PCR-RFLP	0.051	8/9
De Marco P, 2006	Italy	Caucasian	375	523	NTD children, NTD fathers, NTD mothers, healthy controls	PCR-RFLP	0.367	7/9
Parle-McDermott A, 2006	Ireland	Caucasian	548	770	NTD children, NTD fathers, NTD mothers, healthy controls	PCR-RFLP	0.040	6/9
van der Linden IJ, 2007	Netherlands	Caucasian	216	360	NTD cases, NTD mothers, pediatric controls, female controls	PCR-RFLP	0.985	7/9
Carroll N, 2009	Ireland	Caucasian	1391	446	NTD cases, NTD mothers, pediatric controls, female controls	PCR-RFLP	0.027	8/9
Shaw GM, 2009	USA	Caucasian	359	259	NTD cases, healthy controls	PCR-RFLP	0.688	8/9
Marini NJ, 2011	USA	Caucasian	241	239	NTD cases, healthy controls	PCR-RFLP	0.587	8/9
Fisk Green R, 2013	Ireland	Caucasian	13	309	NTD cases, healthy controls	PCR-RFLP	0.968	7/9

PCR-RFLP, Polymerase chain reaction-restriction fragment length polymorphism; PCR-SSCP, PCR-single strand conformation polymorphism; NOS, Newcastle-Ottawa scale.

### Results of the overall meta-analysis

**Figure 2:**
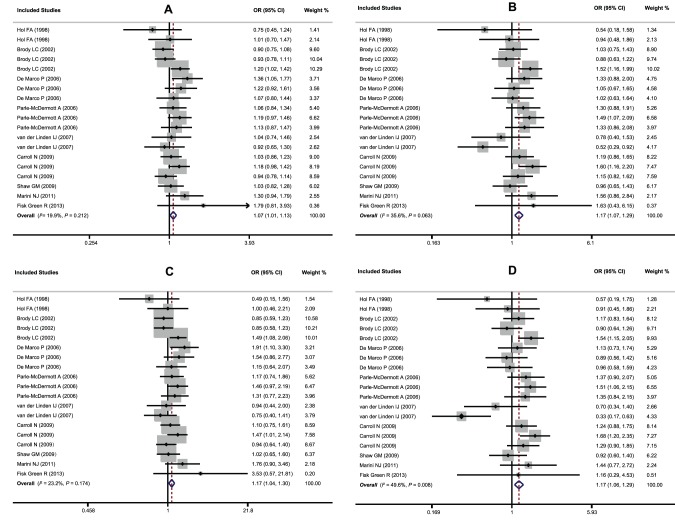
Forest plots of ORs for the association between the *MTHFD1* G1958A polymorphism and susceptibility to NTDs (A: A allele vs. G allele; B: AA vs. AG+GG; C: AA vs. GG; D: AA vs. AG).

Our main results on the association between the *MTHFD1* G1958A polymorphism and NTDs are listed in Table 2. Since no heterogeneity between study-specific effects was observed, the fixed-effect model was conducted in all genetic models except for recessive and heterozygous comparisons, which showed significant heterogeneity (*P*<0.1) When the data from all eligible studies, with a total of 4,302 NTDs cases and 4,238 controls, were pooled together, a borderline significant overall association was found under four genetic models (A allele vs. G allele: OR = 1.07, 95%CI: 1.01–1.13, *P* = 0.014; AA vs. AG+GG: OR = 1.17, 95%CI: 1.07–1.29, *P* = 0.001; AA vs. GG: OR = 1.16, 95%CI: 1.04–1.30, *P* = 0.006; AA vs. AG: OR = 1.17, 95%CI: 1.06–1.29, *P* = 0.002, Figure 2). Sequential removal of each eligible study did not result in movement of the point estimate outside the pooled 95%CIs, indicating no single study had excessive influence on the results of this meta-analysis (data not shown). However, for publication bias, the shape of the Begg’s funnel plot did reveal evidence of obvious asymmetry and Egger’s linear regression test also showed significant publication bias (t = −2.57, *P* = 0.02), indicating that there might be a differential magnitude of effect between large and small studies.

**Table 2:** Summary ors of the association between *MTHFD1* G1958A polymorphism and risk for NTDs.

Genetic models	Study subjects	NO. of studies	OR (95% CI)	*P*-value	Test of heterogeneity		
					*I^2^* (%)	*P-*value	Model
A allele vs. G allele	Overall	9	1.07 (1.01–1.13)	0.014	19.9	0.212	Fixed
	NTD cases	5	1.04 (0.95–1.15)	0.388	28.3	0.233	Fixed
	Mothers	5	1.17 (1.07–1.29)	0.001	0.0	0.716	Fixed
	Fathers	4	0.97 (0.87–1.08)	0.608	0.0	0.470	Fixed
AA+AG vs. GG	Overall	9	1.03 (0.95–1.13)	0.124	28.1	0.418	Fixed
	NTD cases	5	1.03 (0.89–1.20)	0.678	41.0	0.148	Fixed
	Mothers	5	1.15 (0.99–1.34)	0.060	0.0	0.457	Fixed
	Fathers	4	0.86 (0.73–1.10)	0.074	20.7	0.286	Fixed
AA vs. AG+GG	Overall	9	1.17 (1.07–1.29)	0.001	35.6	0.063	Fixed
	NTD cases	5	1.09 (0.92–1.30)	0.289	11.3	0.341	Fixed
	Mothers	5	1.34 (1.15–1.57)	<0.001	71.6	0.007	Fixed
	Fathers	4	1.11 (0.92–1.35)	0.263	0.0	0.797	Fixed
AA vs. GG	Overall	9	1.16 (1.04–1.30)	0.006	23.4	0.174	Fixed
	NTD cases	5	1.10 (0.90–1.35)	0.345	32.8	0.203	Fixed
	Mothers	5	1.39 (1.15–1.68)	0.001	2.0	0.395	Fixed
	Fathers	4	0.99 (0.79–1.23)	0.918	0.0	0.571	Fixed
AA vs. AG	Overall	9	1.17 (1.06–1.29)	0.002	49.6	0.008	Random
	NTD cases	5	1.08 (0.91–1.30)	0.364	12.6	0.333	Random
	Mothers	5	1.30 (1.10–1.54)	0.002	83.1	<0.001	Random
	Fathers	4	1.19 (0.98–1.46)	0.082	0.0	0.755	Random

OR, odd ratio; CI; confidence interval.

### Results of the stratified analysis

**Figure 3:**
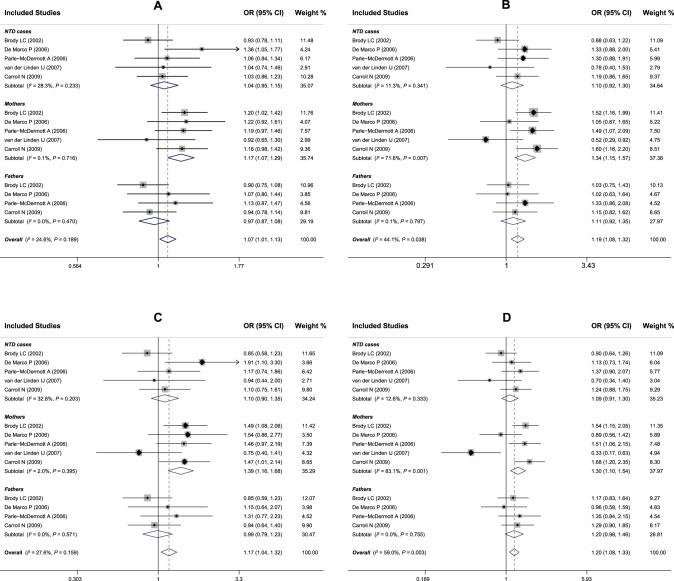
Forest plots of ORs for the association between the *MTHFD1* G1958A polymorphism and susceptibility to NTDs in family-based studies for each subgroup under (A: A allele vs. G allele; B: AA vs. AG+GG; C: AA vs. GG; D: AA vs. AG).

The comparisons of the subgroups in family-based studies are also summarized in Table 2. The fixed-effect model was conducted in all genetic models with the exception of heterozygous comparison. Interestingly, in the stratified analysis, we found that the *MTHFD1* G1958A polymorphism played different roles in different populations. The sample sizes were 1,257/2,939 in NTDs children and controls, 1,356/2,893 in mothers with NTDs offspring and controls, and 962/2,736 in fathers with NTDs offspring and controls. For mothers with NTDs offspring, subjects harboring the 1958A variant are approximately 15–30% more likely to have NTDs when compared to subjects with the 1958G allele (A allele vs. G allele: OR = 1. 17, 95%CI: 1.07–1.29, *P* = 0.001; AA vs. AG+GG: OR = 1. 34, 95%CI: 1.15–1.57, *P* = <0.001; AA vs. GG: OR = 1. 39, 95%CI: 1.15–1.68, *P* = 0.001; AA vs. AG: OR = 1. 30, 95%CI: 1.10–1.54, *P* = 0.002). However, there emerged no evidence of a significant association between the *MTHFD1* G1958A polymorphism and NTDs risk in children and fathers with NTDs offspring under either genetic model (all *P*>0.05) (Figure 3). Since the populations of individual studies were entirely composed of Caucasians, subgroup analysis based on ethnicity was not performed in this meta-analysis.

## Discussion

Neural tube defects (NTDs) are one of the most prevalent and most severe congenital malformations with a high mortality rate. It is widely known and accepted that the pathogenesis of NTDs is unlikely to be ascribed to any single factor, either genetic or environmental. Previous research has revealed that folic acid or folic acid containing multivitamin supplementation in early pregnancy can prevent not only NTDs [4], [33] but also several other congenital anomalies, including orofacial clefts and selected heart defects [34]–[36], to a large extent. Mechanisms underlying these protective effects have not completely been elucidated, but it has been speculated that supplementation with vitamins containing folic acid can overcome disruptions in folate metabolism, which are partially caused by underlying genetic variants involved in the folate metabolic pathway. Therefore, folate-related genes widely investigated for their potential involvement in pathogenesis of NTDs. To date, several meta-analyses have been conducted to investigate the association between NTDs and SNPs for candidate genes involved in the folate pathway, including C677T and A1298C in the *MTHFR* (methylenetetrahydrofolate reductase) gene [37]–[39], A66G in the *MTRR* (methionine synthase reductase) gene [1], [39], A2756G in the *MTR* (methionine synthase) gene [39]–[41], and A80G in the *RFC-1* (reduced folate carrier) gene [39], [42]. However, no meta-analysis has been conducted on the effect of the *MTHFD1* genetic polymorphism on susceptibility to NTDs.

The first report evaluating the use of the *MTHFD1* G1958A polymorphism for predicting NTDs risk was conducted by Hol et al. and proved no evidence for involvement of the G1958A alteration in NTDs etiology [15]. In contrast, several follow-up genetic association studies indicated an association between the G1958A polymorphism and NTDs risk [16]–[18]. These conflicting conclusions might be partly due to relatively limited sample sizes, which can greatly enhance the rate of false-negative and false-positive results. Moreover, the discrepancies between these studies might be the consequence of their use of different selection criteria for NTDs cases and controls. While some only focused on familial and sporadic NTD cases, others concentrated on NTDs cases and parents with NTDs offspring compared with the same controls or different controls in different groups, making their results difficult to interpret. Thus, in the present study, we conducted a comprehensive meta-analysis integrating data from previous publications to derive a more precise assessment of the relationship between the *MTHFD1* G1958A polymorphism and susceptibility to NTDs, which may be helpful in identifying which part of the complex folate metabolism is related to NTDs. To the best of our knowledge, this study is the first meta-analysis to comprehensively assess the association of the G1958A polymorphism with susceptibility to NTDs.

Our results demonstrated a significant correlation between the *MTHFD1* G1958A polymorphism and NTDs in an overall meta-analysis of seven studies. The rationale for the possible association is based on the enzyme activities of *MTHFD1* that play a central role in folate pool maintenance. It is also possible that this mutation may be in linkage disequilibrium with another, as yet undescribed, variant that alters function. In this meta-analysis, we also performed a stratified analysis for the five family-based studies by classifying study subjects as NTD cases, mothers with NTDs offspring, and fathers with NTDs offspring. As for the NTDs cases subgroup, no significant allelic or genotypic association between this polymorphism and NTDs could be observed. However, for the parents with NTDs offspring subgroups, a comparison between groups yielded significant evidence for the overrepresentation of the 1958A allele and AA homozygote among the case mothers, compared with control individuals, suggesting that the AA genotype may have a different effect in the embryo and may have a maternal effect only. It is noteworthy that women who harbor the AA homozygote for the *MTHFD1* G1958A polymorphism are almost three times more likely to develop severe abruptio placentae during their pregnancy than women who are ‘GA’ or ‘GG’ [43]. In addition, Parle-McDermott et al. also reported that *MTHFD1* 1958AA homozygote have a 1.64-fold increased risk of having an unexplained second trimester loss, which suggests that the *MTHFD1* 1958AA genotype may be an important maternal risk factor to consider during pregnancy [44]. Thus, it can be hypothesized that the AA genotype of the G1958A polymorphism may produce more severely affected NTDs embryos that do not survive to birth. Since the family-based studies were less powerful than the case-control studies, these results should be interpreted with caution and need to be confirmed in further studies.

Meta-analysis is a useful method in synthesizing data from all the eligible studies to obtain greater statistical power. However, several specific issues in this meta-analysis should be considered. First, our study was based on single-factor estimates without adjustment for other risk factors since no information on potential confounders was obtained, a fact that might have caused bias. Second, all the study populations included in our meta-analysis were Caucasian and thus we couldn’t perform stratified analyses based on ethnicity. Hence, for Asian and African populations, the association of the *MTHFD1* G1958A polymorphism with susceptibility to NTDs should be interpreted in caution, and more large-scale studies on other ethnicities are warranted to support our findings. Third, NTD defects cover a continuum of differing severity and the outcome of an NTD patient varies from livebirth to stillbirth, and thus the effects of genetic variants on risk of NTDs may be underestimated if studies only collect livebirths and less severity cases. Last, given that the significant publication bias was found in this meta-analysis and the nature of publication of genetic association studies, our results should be interpreted with caution. However, notwithstanding the preceding limitations, our meta-analysis also had some strengths: (1) we analyzed the association between the *MTHFD1* G1958A polymorphism and the NTDs risk in three groups (NTDs patients, mothers, and fathers), which minimized the influence of confounding factors; (2) sensitivity analysis did not show any single study strongly affecting the combined results, suggesting sufficient reliability and stability of the pooled results; (3) this is the first meta-analysis to combine data from previous studies on relationship between the *MTHFD1* G1958A polymorphism and NTDs pathogenesis.

In summary, the current meta-analysis demonstrated a significant correlation between the *MTHFD1* G1958A and an increased risk of NTDs, especially in mothers of children with NTDs. However, due to the limitations of this study and the low edge of 95%CI, which nearly touched the null value, these results should be viewed with caution. Further large-scale studies will be needed to provide more conclusive and accurate evidence regarding relationships between SNPs in the folate pathway genes and NTDs and how these relationships impact women during the crucial period of pregnancy.

## Supporting Information

Supplement S1 PRISMA 2009 Checklist.
(DOC)

Supplement S2 Newcastle-Ottawa Quality Assessment Scale.
(DOC)

Supplement S3 **Full-text articles excluded (a); Studies only included in qualitative synthesis (b).**
(DOC)
