# Supplementary material for: Association between MTHFD1 G1958A Polymorphism and Neural Tube Defects Susceptibility: A Meta-Analysis
Source: PLoS One. 2014 Jun 30;9(6):e101169. doi: 10.1371/journal.pone.0101169 (PMC4076264; doi:10.1371/journal.pone.0101169)
Supplement: Supplement S3 — Full-text articles excluded (a); Studies only included in qualitative synthesis (b). (DOC) [file pone.0101169.s003.doc]

**(a) Full-text articles excluded (n = 4)**

| First author, year (PMID) | Reason for exclusion |
| --- | --- |
| Mills JL, 2005 (15937947) | Focusing on patients with omphalocele |
| Boyles AL, 2006 (17035141) | A case-only study |
| Parle-McDermott A, 2009 (19777576) | Focusing on the rs3832406 variant in *MTHFD1L* gene |
| Minguzzi S, 2012 (22520921) | Focusing on the rs3832406 variant in *MTHFD1L* gene |

**(b) Studies only included in qualitative synthesis (n = 3)**

| First author, year (PMID) | Reason for exclusion |
| --- | --- |
| Etheredge AJ, 2012 (22903727) | Do not provide sufficient data for each genotype |
| Pangilinan F, 2012 (22856873) | Do not provide sufficient data for each genotype |
| Liu J, 2014 (24326202) | Do not provide sufficient data for each genotype |
